# Animal foods, protein, calcium and prostate cancer risk: the European Prospective Investigation into Cancer and Nutrition

**DOI:** 10.1038/sj.bjc.6604331

**Published:** 2008-04-01

**Authors:** N E Allen, T J Key, P N Appleby, R C Travis, A W Roddam, A Tjønneland, N F Johnsen, K Overvad, J Linseisen, S Rohrmann, H Boeing, T Pischon, H B Bueno-de-Mesquita, L Kiemeney, G Tagliabue, D Palli, P Vineis, R Tumino, A Trichopoulou, C Kassapa, D Trichopoulos, E Ardanaz, N Larrañaga, M-J Tormo, C A González, J R Quirós, M-J Sánchez, S Bingham, K-T Khaw, J Manjer, G Berglund, P Stattin, G Hallmans, N Slimani, P Ferrari, S Rinaldi, E Riboli

**Affiliations:** 1 Cancer Epidemiology Unit, University of Oxford, Oxford, UK; 2 Institute of Cancer Epidemiology, Danish Cancer Society, Copenhagen, Denmark; 3 Department of Epidemiology and Social Medicine, University of Aarhus, Aarhus, Denmark; 4 Division of Cancer Epidemiology, German Cancer Research Centre, Heidelberg, Germany; 5 German Institute of Human Nutrition, Potsdam-Rehbücke, Germany; 6 National Institute of Public Health and Environment, Bilthoven, The Netherlands; 7 Department of Epidemiology, University Medical Centre, Nijmegen, The Netherlands; 8 Department of Urology, University Medical Centre, Nijmegen, The Netherlands; 9 Lombardy Cancer Registry Unit, National Cancer Institute, Milan, Italy; 10 Molecular and Nutritional Epidemiology Unit, Scientific Institute of Tuscany, Florence, Italy; 11 Department of Biomedical Science, University of Turin, Turin, Italy; 12 Department of Epidemiology and Public Health, Imperial College, London, UK; 13 Cancer Registry, Azienda Ospedaliera Civile M.P. Arezzo, Ragusa, Italy; 14 Department of Hygiene and Epidemiology, University of Athens Medical School, Athens, Greece; 15 Hellenic Health Foundation, Athens, Greece; 16 Seccion de Vigilancia y Control Epidemiologico, Instituto de Salud Publica de Navarra, Pamplona, Spain; 17 Epidemiology Unit, Basque Health Department in Gipuzkoa, San Sebastian, Spain; 18 Epidemiology Department, Murcia Health Council, Murcia, Spain; 19 Catalan Institute of Oncology, Barcelona, Spain; 20 Public Health and Health Planning Directorate, Asturias, Spain; 21 Andalusian School of Public Health, Granada, Spain; 22 MRC Centre for Nutritional Epidemiology in Cancer Prevention and Survival, Department of Public Health and Primary Care, Cambridge, UK; 23 Department of Gerontology, University of Cambridge, Cambridge, UK; 24 Department of Surgery, Malmö University Hospital, Lund University, Malmö, Sweden; 25 Department of Clinical Sciences, Malmö University Hospital, Lund University, Malmö, Sweden; 26 Division of Urology and Andrology, Department of Surgical and Perioperative Sciences, Umeå University Hospital, Umeå, Sweden; 27 Department of Public Health and Clinical Medicine, Nutrition Research, Umeå University Hospital, Umeå, Sweden; 28 Nutrition and Hormones Group, International Agency for Research on Cancer, Lyon, France

**Keywords:** prostate cancer, dairy protein, calcium, prospective, EPIC

## Abstract

We examined consumption of animal foods, protein and calcium in relation to risk of prostate cancer among 142 251 men in the European Prospective Investigation into Cancer and Nutrition. Associations were examined using Cox regression, stratified by recruitment centre and adjusted for height, weight, education, marital status and energy intake. After an average of 8.7 years of follow-up, there were 2727 incident cases of prostate cancer, of which 1131 were known to be localised and 541 advanced-stage disease. A high intake of dairy protein was associated with an increased risk, with a hazard ratio for the top *versus* the bottom fifth of intake of 1.22 (95% confidence interval (CI): 1.07–1.41, *P*_trend_=0.02). After calibration to allow for measurement error, we estimated that a 35-g day^−1^ increase in consumption of dairy protein was associated with an increase in the risk of prostate cancer of 32% (95% CI: 1–72%, *P*_trend_=0.04). Calcium from dairy products was also positively associated with risk, but not calcium from other foods. The results support the hypothesis that a high intake of protein or calcium from dairy products may increase the risk for prostate cancer.

Little is known about the aetiology of prostate cancer. It has been suggested that a high intake of animal protein might increase the incidence of prostate cancer by enhancing growth hormone activity (Sato, 1963). Ecological studies have shown that milk intake is strongly correlated with both incidence and mortality from prostate cancer (Ganmaa *et al*, 2002; Colli and Colli, 2006). It has been hypothesised that a high intake of dairy protein may increase prostate cancer risk by increasing the production of insulin-like growth-factor-I (IGF-I), which in turn may promote development of prostate cancer (Renehan *et al*, 2004; Allen *et al*, 2007). An alternative hypothesis is that a high intake of calcium, primarily from dairy products, may increase risk by suppressing the synthesis of 1,25-dihydroxyvitamin D (Giovannucci, 1998).

In the present study, we investigated prostate cancer risk in relation to consumption of animal foods, protein and calcium among 142 520 men in the European Prospective Investigation into Cancer and Nutrition (EPIC).

## MATERIALS AND METHODS

The European Prospective Investigation into Cancer and Nutrition is a multicentre prospective study designed to investigate the relationships between diet, lifestyle, environmental factors and cancer. The methods of recruitment and study design are fully described elsewhere (Riboli *et al*, 2002). The total cohort comprises approximately 500 000 men and women recruited in 28 centres in 10 European countries: Denmark, France, Germany, Greece, Italy, the Netherlands, Norway, Spain, Sweden and the United Kingdom (UK). In this paper, we describe data for men from 19 centres in eight of these countries, no data being available for France or Norway because only women were recruited in these two countries. Men were recruited between 1989 and 2004, and the median age at recruitment was 52 years.

The men included in this analysis were recruited from the population of defined geographical areas in each of the eight countries (general population in most centres, blood donors in Ragusa and Turin in Italy and in the Spanish centres), except for most of those in the Oxford subcohort, who were recruited throughout the United Kingdom to enroll a large number of vegetarians. Study participants were almost all white Europeans. Eligible men were invited to participate in the study, and those who accepted gave informed consent and completed questionnaires on their diet, lifestyle and medical history. Approval for this study was obtained from the ethical review boards of the International Agency for Research on Cancer (IARC) and from local ethics committees in each country.

Men were not eligible for this analysis if they had previously been registered as having cancer at the time of completing the baseline questionnaire, if they had no dietary or nondietary data, or if they had missing dates of cancer diagnosis or follow-up. Individuals were also excluded if they were in the top or bottom 1% of the distribution of the ratio of reported energy intake to energy requirement, to reduce the impact of implausible extreme values in the analysis (Ferrari *et al*, 2002). Following these exclusions, complete data on diet and follow-up for cancer were available for 142 520 men out of the 148 372 men in the original data set.

Dietary intake during the year before enrolment was measured by country-specific validated food frequency questionnaires (FFQs) or diet histories, as previously described (Riboli *et al*, 2002). For this analysis, animal foods included total meat and meat products (and the subcategories red meat, poultry and processed meat), fish and shellfish (and the subcategories white fish and fatty fish), dairy products (and the subcategories milk and milk beverages, yoghurt and cheese) and eggs. Estimated daily nutrient intakes were calculated by multiplying the nutrient content of each food of a specific portion size by the frequency of consumption as stated on the FFQ using national food tables from each country. Greece and Umeå were not included in the analyses of animal, dairy or plant protein, or Greece in that of calcium intake, because the relevant data were not available.

The nondietary questions covered education and socioeconomic status, occupation, history of previous illness and disorders or surgical operations, lifetime history of consumption of tobacco and alcoholic beverages, and physical activity. Height and weight were measured at recruitment, except for most of the men in the Oxford cohort among whom height and weight were self-reported.

Follow-up is provided by population-based cancer registries in six of the participating countries: Denmark, Italy, the Netherlands, Spain, Sweden and the United Kingdom. In Germany and Greece, follow-up is via self-completed questionnaires, and self-reported incident cancers are verified through medical records. Data on vital status in most EPIC centres were collected from mortality registries at the regional or national level, in combination with data collected by active follow-up (Greece). By March 2007, complete follow-up data had been reported to IARC up to December 2003 or December 2004 for most centres. Follow-up was censored at the date of diagnosis of prostate cancer, or at the date of diagnosis of other cancers, death, emigration or end of follow-up, whichever came first. The 10th Revision of the International Statistical Classification of Diseases, Injuries and Causes of Death (ICD) was used, and cancer of the prostate as analysed here was defined as code C61.

Data on TNM stage and Gleason grade were collected from each centre, where possible. In all, 1672 cases (61%) had information on stage and 1630 cases (60%) had information on grade. Tumours were classified as localised (TNM staging score of T0/T1/T2 and N0/NX and M0, or stage coded in the recruitment centre as localised; *n*=1131) or advanced (T3 or T4 or N1+ or M1, or stage coded in the recruitment centre as metastatic; *n*=541) or unknown. Subset analyses were also conducted for low-grade (Gleason sum <7 or equivalent (cases coded as well differentiated or moderately differentiated); *n*=982) and high-grade disease (Gleason sum ⩾7 or equivalent (cases coded as poorly differentiated or undifferentiated); *n*=648), or unknown.

### Statistical analyses

Analyses of the associations of foods and nutrients and potential confounding factors with risk were conducted using Cox regression. Data were stratified by centre, and age was used as the underlying time scale in all models.

Food and nutrient intakes estimated from the dietary questionnaires (Riboli *et al*, 2002) were calculated in g day^−1^, unless otherwise stated. Dietary intakes were primarily analysed as categorical variables, based on quintiles of the distribution among noncases across all EPIC centres combined. Tests for linear trend were conducted using continuous values for each food and nutrient variable; nutrient increments were set at 35 g day^−1^ for all protein variables and 0.3 g day^−1^ for all calcium variables (corresponding to approximately 1 s.d. in total protein and calcium intake, respectively) to make comparable assessments of intake with risk. All models were adjusted for educational level (no degree, degree or higher, unknown), marital status (married/cohabiting, not married/cohabiting and unknown), height (<170, 170–174, 175–179 and ⩾180 cm), weight (<70, 70–79, 80–89 and ⩾90 kg) and energy intake (MJ day^−1^; continuous). Further adjustment for combined occupational and recreational physical activity (physically inactive, moderately inactive, moderately active, active and not known) smoking (never, past, current and unknown) and alcohol (continuous) made no appreciable difference to the results so these covariates were not included in the final models.

To improve the comparability of dietary data across participating centres and to correct for measurement error in relative risk estimates, dietary intakes from the questionnaires were calibrated using a fixed-effects linear model in which centre and gender-specific 24-h recall data from an 8% random sample of the cohort (Slimani *et al*, 2002) were regressed on the FFQ intakes (Ferrari *et al*, 2007).

Separate analyses were conducted for localised and advanced disease, and also for low-grade and high-grade disease. Heterogeneity between these subgroups was tested by fitting stratified Cox models based on competing risks, comparing the risk coefficients and standard errors in the subgroups of interest after excluding cases of uncertain stage and grade as previously described (Smith-Warner *et al*, 2006). To evaluate whether preclinical disease may have influenced results, additional analyses were conducted after excluding the first 4 years of follow-up. We also examined whether the association between animal foods and risk was modified by age at recruitment (<60 and ⩾60 years). Heterogeneity in the association with prostate cancer risk between countries was assessed using *χ*^2^ tests. All *P*-values presented are two-tailed and *P*-values below 0.05 were considered statistically significant. Analyses were performed using Stata v. 9 (Stata Corporation, Texas, USA).

## RESULTS

Details of participants from the eight countries are shown in Table 1. After an average of 8.7 years of follow-up, 2727 men were diagnosed with prostate cancer among the 142 520 participants included in this study, with a total of 1 236 265 person-years. The median age at diagnosis of prostate cancer was 66 years (range: 44–95 years). There was an approximate two-fold variation in the calibrated median intake of total meat and meat products among participating countries, and a three- to six-fold variation in the intake of red meat, poultry, milk and milk beverages, cheese and eggs; the median intake of processed meat and yoghurt varied by more than 10-fold among countries. On the basis of 24-h recall data, protein intake was largely derived from meat (32%), cereals (18%), cheese (9%) and milk (7%). Overall, 17% of protein was derived from dairy products, although this varied from 11% in Spain to 23% in Sweden. Dietary calcium was largely derived from dairy products (53%), with milk, cheese and yoghurt contributing 22, 23 and 8%, respectively. Variation in the proportion of calcium derived from dairy foods ranged from 43% in Germany to 65% in Sweden. There was a very strong correlation between dairy protein and dairy calcium intake (*r*=0.98).

Differences in nondietary characteristics at baseline between prostate cancer cases and noncases are shown in Table 2. Cases were older, less likely to be current smokers or physically active, and were more likely to be married than noncases. In Cox regression analyses, being educated to a degree level or higher, and being married or cohabiting, were each associated with a statistically significant increased risk (results not shown) and were therefore included in subsequent models of dietary intake and risk.

Table 3 shows hazard ratios (HRs) for prostate cancer in relation to consumption of meat, fish, dairy foods and eggs, stratified by centre and adjusted for education, marital status, height, weight and energy intake. Overall, there was no association between any of the meat or fish products or eggs and risk. For dairy products, yoghurt intake was associated with an increased risk (the HR for the highest *versus* the lowest fifth of intake was 1.17, 95% confidence interval (CI): 1.04–1.31; *P*_trend_=0.02), but there was no evidence of an association with intakes of milk and milk beverages or cheese.

**Figure 1:**
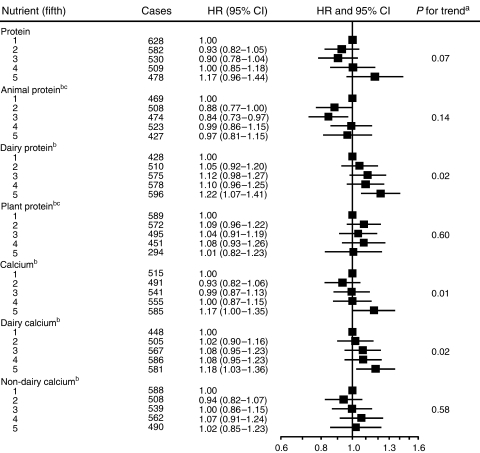
Multivariate HRs and 95% CIs for prostate cancer by quintile of observed intake of protein and calcium. All models are stratified by centre and adjusted for education, marital status, height, weight and energy intake. Mean intakes in each quintile based on 24-h recall data are: 80, 90, 98, 105 and 121 g day^−1^ for protein; 47, 59, 64, 69 and 80 g day^−1^ for animal protein; 10, 14, 17, 21 and 27 g day^−1^ for dairy protein; 29, 33, 36, 38 and 47 g day^−1^ for plant protein; 0.78, 0.92, 1.01, 1.10 and 1.32 g day^−1^ for total calcium; 0.30, 0.44, 0.56, 0.68 and 0.88 g day^−1^ for dairy calcium; and 0.38, 0.42, 0.45, 1.8 and 5.5 g day^−1^ for nondairy calcium. ^a^*P*-values for trend are obtained by entering the continuous variable in the model. ^b^Greece excluded. ^c^Umeå excluded.

Total protein intake was nonsignificantly positively associated with increased risk (HR in the highest *versus* the lowest fifth of intake=1.17, 95% CI: 0.96–1.44; *P*_trend_=0.07) (Figure 1). Protein from dairy foods was significantly associated with an increased risk (HR for the highest *versus* lowest fifth was 1.22, 95% CI: 1.07–1.41; *P*_trend_=0.02). Protein derived from all animal or all plant foods was not significantly associated with risk. Total dietary calcium intake and calcium intake from dairy foods were also associated with an increased risk (HR for the highest *versus* the lowest fifth of intake were 1.17, 95% CI: 1.00–1.35; *P*_trend_=0.01 for total dietary calcium, and 1.18, 95% CI: 1.03–1.36; *P*_trend_=0.02 for dairy calcium). Calcium intake from nondairy foods was not associated with risk.

Table 4 shows the HRs for prostate cancer associated with consumption of protein and calcium evaluated as continuous variables, before and after calibration. Protein intake from dairy products, total dietary calcium and calcium from dairy products were all associated with a significant increase in risk, which was larger after calibration. An increment of 35 g day^−1^ dairy protein was associated with an HR of 1.32 (95% CI: 1.01–1.72; *P*_trend_=0.04) and increments of 0.3 g day^−1^ of total calcium and dairy calcium were associated with HRs of 1.09 (95% CI: 1.01–1.16; *P*_trend_=0.02) and 1.07 (95% CI: 1.00–1.14; *P*_trend_=0.04), respectively.

The associations between calibrated estimates of protein and calcium intake and risk according to stage and grade of disease are shown in Table 5. Risk estimates for most nutrients were slightly higher for localised disease (*n*=1131) than advanced disease (*n*=541), but none reached statistical significance. No nutrients were associated with low-grade disease, but intakes of dairy protein, total dietary calcium and dairy calcium were significantly associated with an increased risk of high-grade disease (HRs of 1.76, 95% CI: 1.06–2.95; *P*_trend_=0.03 for an increment increase of 35 g day^−1^ dairy protein; and 1.19, 95% CI: 1.04–1.37; *P*_trend_=0.01 and 1.16, 95% CI: 1.03–1.32; *P*_trend_=0.02 for an increment increase of 0.3 g day^−1^ of total calcium and dairy calcium, respectively). However, the tests for heterogeneity between the risk estimates for low-grade and high-grade disease were not statistically significant for any of these nutrients.

The associations between nutrients and prostate cancer risk were similar below the age of 60 years at recruitment (*n*=1222) and at older ages (*n*=1505), although some significant associations were found for calibrated intakes among men below the age of 60 years at recruitment (HR=1.43, 95% CI: 1.02–2.03, for 35 g day^−1^ of dairy protein, HR=1.10, 95% CI: 1.01–1.20, for 0.3 g day^−1^ for both total and dairy calcium). To examine whether cancers diagnosed soon after recruitment may have influenced the results, the analyses were repeated after excluding the first 4 years of follow-up, leaving 2010 cases and 134 944 noncases, but they did not materially change (results not shown). There was no evidence of heterogeneity between countries in the association of protein or calcium intake with risk of overall prostate cancer (results not shown).

## DISCUSSION

In this prospective study of 2727 cases of prostate cancer, the consumption of protein and calcium derived from dairy foods were significantly positively associated with risk. Strengths of the EPIC study are its prospective design, the large number of prostate cancer cases and the wide range of animal food intakes. We were also able to consider other possible risk factors such as education, marital status, alcohol intake, height, weight, energy intake, smoking and physical activity. Data on PSA use in the EPIC cohort are not available, but the annual rates of PSA testing in middle aged men within some of the participating countries suggest relatively low rates, of 6% in England and Wales, 7% in the Netherlands, 9% in Spain and 16% in Italy, compared with approximately 38% in white Americans (Etzioni *et al*, 2002; Paez *et al*, 2002; Otto *et al*, 2003; D'Ambrosio *et al*, 2004; Melia *et al*, 2004).

Our results are compatible with the hypothesis that a high intake of dairy protein is associated with increased prostate cancer risk (Gao *et al*, 2005). Although we found no association with milk intake *per se*, protein and calcium intake derived from dairy foods, and also yoghurt intake, were significantly associated with increased risk. This apparent discrepancy may, in part, be because dairy protein and calcium are derived from a combination of dairy products, which on their own only exhibit a weak association with risk.

It has been hypothesised that the high protein content of dairy products may increase risk by increasing circulating levels of IGF-I, as shown in several cross-sectional studies (Holmes *et al*, 2002; Giovannucci *et al*, 2003; Heald *et al*, 2003; Larsson *et al*, 2005; Norat *et al*, 2007) and some intervention trials (Heaney *et al*, 1999; Hoppe *et al*, 2004). Vegan men and women (who consume no dairy or other animal products) have significantly lower serum IGF-I levels than both lacto-ovo vegetarians and meat eaters (Allen *et al*, 2000, 2002), which may be due to their lower intake of essential amino acids (Allen *et al*, 2002). Intervention studies have also shown that protein restriction can lower IGF-I levels in both animals (Miura *et al*, 1992; Takenaka *et al*, 2000; Katsumata *et al*, 2002) and humans (Smith *et al*, 1995), and that the increases in IGF-I levels following re-feeding are strongly related to the essential amino-acid component of the diet (Clemmons *et al*, 1985). The effects of protein and especially dairy protein on IGF-I could be important, because high serum IGF-I levels have been associated with a moderately increased risk of prostate cancer in several large-scale prospective studies including EPIC (Renehan *et al*, 2004; Allen *et al*, 2007). However, few studies have reported on protein intake in relation to risk (Severson *et al*, 1989; Schuurman *et al*, 1999; Chan *et al*, 2000), and, to our knowledge, this is the first study to examine specifically the association of dairy protein in risk.

An alternative hypothesis is that dairy products may increase prostate cancer risk via their high calcium content, and our finding of a positive association with calcium intake is consistent with some (Chan *et al*, 2001; Gao *et al*, 2005; Tseng *et al*, 2005; Giovannucci *et al*, 2006; Kesse *et al*, 2006; Ahn *et al*, 2007; Mitrou *et al*, 2007), but not all, prospective studies (Koh *et al*, 2006; Severi *et al*, 2006; Rohrmann *et al*, 2007). It has been suggested that a high calcium intake may increase risk by suppressing the synthesis of 1,25-dihydroxyvitamin D, which has an antitumour effect on human prostatic cells *in vitro* (Giovannucci, 1998). However, a randomised controlled trial showed that, although long-term calcium supplementation (1.2 g calcium per day) slightly reduced serum 1,25-dihydroxyvitamin D concentrations, it was not associated with increased risk, although this trial was too small to exclude a moderate effect (upper CI in treated group 1.32; Baron *et al*, 2005). In addition, the evidence that circulating levels of either 1,25-dihydroxyvitamin D or its precursor 25-dihydroxyvitamin D are inversely related to risk is inconsistent (Giovannucci, 2005). Overall, the evidence that a high calcium intake affects risk through mechanisms related to vitamin D production appears limited. It is possible that the association seen with dairy calcium intake may be due to its high correlation with other aspects of dairy foods, particularly protein, although it is difficult to separate out their independent effects. Further, although the intake of nondairy calcium was low, the finding that it was not associated with risk suggests that another component of dairy foods may be of greater aetiological relevance.

Some studies have suggested that the associations with dairy foods and calcium intake are stronger for aggressive disease (Gao *et al*, 2005; Giovannucci *et al*, 2006). In the current study, although these associations were slightly stronger for high-grade disease and for men recruited before the age of 60 years, the differences between the groups were not significant, and there were no comparable differences between localised and advanced-stage disease. Several previous studies have found that risk is particularly elevated in men with a total calcium intake (i.e., diet plus supplement intake) above 2 g day^−1^ (Rodriguez *et al*, 2003; Giovannucci *et al*, 2006; Ahn *et al*, 2007; Mitrou *et al*, 2007). However, we had no information on supplement use and although the present study found a significantly elevated risk in the top fifth of dietary intake, the mean intake of this category, based on 24-h recall data, was 1.3 g day^−1^; less than 1% of the cohort had a dietary calcium intake of 2 g day^−1^ or more. As such, we had limited power to assess whether very high calcium intake is associated with increased risk.

Our finding that total meat or red meat intake is not associated with prostate cancer risk is consistent with most previous prospective studies (Snowdon, 1988; Mills *et al*, 1989; Hsing *et al*, 1990; Chan *et al*, 2000; Allen *et al*, 2004; Cross *et al*, 2005; Rohrmann *et al*, 2007), although some found positive associations with red meat (Michaud *et al*, 2001), hamburgers (Veierod *et al*, 1997), beef (Le Marchand *et al*, 1994) or cooked processed meat (Rodriguez *et al*, 2006) for either total or advanced prostate cancer. For fish, our results, which are based on a wide range of intake, provide no evidence that intake is associated with risk, and is consistent with most previous studies (Severson *et al*, 1989; Hsing *et al*, 1990; Le Marchand *et al*, 1994; Gronberg *et al*, 1996; Schuurman *et al*, 1999), although some have reported a negative (Terry *et al*, 2001; Augustsson *et al*, 2003) or positive association (Mills *et al*, 1989; Allen *et al*, 2004).

Our study has some limitations. As in other large epidemiological studies, dietary intake is estimated using relatively simple dietary questionnaires that are subject to measurement errors leading to attenuated risk estimates. Nonetheless, the questionnaires in all EPIC centres were validated, and dietary intakes were calibrated with measures from a standardised 24-h diet recall method.

In conclusion, the results from this large prospective study are consistent with the hypothesis that a high intake of protein or calcium from dairy products may increase prostate cancer risk.

## Figures and Tables

**Table 1:** Description of the study cohorts with men participating in the European Prospective Investigation into Cancer and Nutrition (EPIC)

**Country**	**Number of men**	**Cases (nos.)**	**Localised (nos.)**	**Advanced (nos.)**	**Person-years**	**Median age at recruitment (5th–95th percentile)**
Denmark	26 267	368	188	104	198 584	56 (50–64)
Germany	21 567	420	240	92	176 902	52 (41–63)
Greece	10 593	40	17	8	73 980	52 (33–72)
Italy	14 009	145	27	30	120 984	49 (38–62)
The Netherlands	9782	59	11	29	82 852	43 (23–58)
Spain	15 150	206	144	23	156 070	49 (40–63)
Sweden	22 299	1013	323	157	233 424	51 (30–68)
UK	22 853	476	181	98	193 469	52 (28–73)
						
All countries	142 520	2727	1131	541	1 236 265	52 (33–67)

**Table 2:** Baseline nondietary characteristics of prostate cancer cases and noncases in EPIC

**Characteristic**	**Cases**	**Noncases**
Number of subjects	2727	139 793
Age at recruitment (years)^a^	60.3 (6.7)	51.5 (10.1)
Height (cm)^a^	174.3 (7.0)	174.7 (7.3)
Weight (kg)^a^	80.2 (11.4)	80.9 (12.0)
BMI (kg m^−2^)^a^	26.4 (3.4)	26.5 (3.6)
		
*Smoking*, %		
Never	33.6	32.9
Former	43.8	36.1
Current	21.5	29.6
Unknown	1.1	1.4
		
*Combined total physical activity,* %^a^		
Inactive	14.6	18.5
Moderately inactive	30.5	26.2
Moderately active	36.5	32.5
Active	6.9	12.3
Unknown	11.5	10.5
		
*Education,* %		
Below degree level	71.1	70.8
Degree level	25.0	26.2
Unknown	3.9	3.0
		
*Marital status,* %		
Married	65.8	55.8
Not married	12.0	13.2
Unknown	22.2	31.0

a Values are means (and s.d. in parentheses).

**Table 3:** Multivariate hazard ratios (HRs) and 95% confidence intervals (CIs) for prostate cancer among 142 520 men in the EPIC cohort by fifths of observed intake of meat, fish and dairy foods

	**Fifth of food intake**										
	**1**		**2**		**3**		**4**		**5**		
**Food**	**No. of cases**	**HR (95% CI)**	**No. of cases**	**HR (95% CI)**	**No. of cases**	**HR (95% CI)**	**No. of cases**	**HR (95% CI)**	**No. of cases**	**HR (95% CI)**	***P*_trend_ a**
*Meat and meat products* b	442	1.00 (referent)	601	1.08 (0.95–1.23)	512	0.96 (0.84–1.10)	473	0.99 (0.86–1.15)	413	0.97 (0.83–1.14)	0.85
Red meat^b^	499	1.00 (referent)	602	1.03 (0.91–1.17)	532	1.00 (0.88–1.13)	437	0.97 (0.84–1.12)	371	0.96 (0.82–1.12)	0.69
Poultry	738	1.00 (referent)	534	1.05 (0.93–1.18)	517	1.11 (0.98–1.25)	471	1.07 (0.95–1.21)	467	1.12 (0.98–1.27)	0.06
Processed meat	345	1.00 (referent)	586	1.06 (0.92–1.22)	577	1.03 (0.89–1.19)	629	1.03 (0.89–1.20)	590	0.93 (0.79–1.09)	0.54
											
*Fish and fish products*	431	1.00 (referent)	464	1.00 (0.87–1.14)	582	1.09 (0.96–1.25)	585	1.02 (0.90–1.17)	665	1.05 (0.91–1.20)	0.56
White fish^c^	436	1.00 (referent)	260	1.08 (0.91–1.27)	319	0.97 (0.83–1.13)	452	0.99 (0.86–1.14)	554	1.03 (0.90–1.18)	0.90
Fatty fish	579	1.00 (referent)	382	1.04 (0.91–1.19)	562	1.05 (0.94–1.19)	538	1.03 (0.92–1.17)	666	1.07 (0.95–1.21)	0.95
											
*Milk and milk beverages*	470	1.00 (referent)	489	1.00 (0.88–1.14)	517	0.98 (0.86–1.11)	594	0.97 (0.85–1.10)	657	1.01 (0.89–1.16)	0.23
Yoghurt	684	1.00 (referent)	351	0.91 (0.79–1.06)	434	1.08 (0.94–1.23)	516	1.09 (0.96–1.23)	742	1.17 (1.04–1.31)	0.02
Cheese	552	1.00 (referent)	584	0.98 (0.87–1.11)	575	1.00 (0.89–1.14)	591	1.10 (0.97–1.24)	425	1.04 (0.90–1.20)	0.58
											
Eggs^b^	518	1.00 (referent)	474	0.98 (0.87–1.11)	441	0.98 (0.86–1.12)	514	0.99 (0.87–1.12)	494	0.96 (0.84–1.10)	0.33

All models are stratified by centre and adjusted for education, marital status, height, weight and energy intake. Median intakes in each fifth based on 24-h recall data are: 76, 119, 140, 162 and 194 g day^−1^ for total meat and meat products; 28, 50, 61, 72 and 90 g day^−1^ for red meat; 9, 13, 19, 22 and 32 g day^−1^ for poultry; 16, 42, 53, 71 and 88 g day^−1^ for processed meat; 18, 29, 38, 49 and 78 g day^−1^ for fish and fish products; 13, 13, 17, 25 and 43 g day^−1^ for white fish; 9, 13, 16, 20 and 32 g day^−1^ for fatty fish; 34, 90, 200, 265 and 466 g day^−1^ for milk and milk beverages; 12, 10, 26, 59 and 135 g day^−1^ for yoghurt (also includes fromage blanc and petits suisses); 15, 28, 35, 40 and 57 g day^−1^ for cheese; and 9, 12, 15, 20 and 32 g day^−1^ for eggs.

a *P*-values for trend are obtained by entering the continuous food intake variable in the model.

b Umeå excluded due to missing data.

c Germany and Umeå excluded due to missing data.

**Table 4:** Multivariate hazard ratios (HRs) and 95% confidence intervals (CIs) for prostate cancer among 142 520 men in the EPIC cohort for specified increments in observed (uncalibrated) and calibrated intakes of protein and calcium

	**Increment**	**Observed**		**Calibrated**	
**Foods and nutrients**	**(g day^−1^)**	**HR (95% CI)**	***P*_trend_**	**HR (95% CI)**	***P*_trend_**
*Protein*	35	1.09 (0.99–1.19)	0.07	1.24 (0.97–1.59)	0.08
Animal protein^a^	35	1.07 (0.98–1.17)	0.14	1.07 (0.89–1.29)	0.47
Dairy protein^b^	35	1.16 (1.03–1.31)	0.02	1.32 (1.01–1.72)	0.04
Plant protein^a^	35	0.95 (0.77–1.17)	0.60	0.94 (0.59–1.49)	0.80
					
*Calcium* b	0.3	1.04 (1.01–1.08)	0.01	1.09 (1.01–1.16)	0.02
Dairy calcium^b^	0.3	1.04 (1.01–1.08)	0.02	1.07 (1.00–1.14)	0.04
Nondairy calcium^b^	0.3	1.04 (0.90–1.19)	0.58	1.18 (0.83–1.68)	0.36

All models are stratified by centre and adjusted for education, marital status, height, weight and energy intake.

a Greece and Umeå excluded due to missing data.

b Greece excluded due to missing data.

**Table 5:** Multivariate hazard ratios (HRs) and 95% confidence intervals (CIs) by stage and grade of prostate cancer for specified increments in calibrated intakes of protein and calcium in EPIC

		**Localised stage disease (*n*=1403)**		**Advanced-stage disease (*n*=541)**			**Low-grade disease (*n*=982)**		**High-grade disease (*n*=648)**		
**Nutrient**	**Increment (g day^−1^)**	**HR (95% CI)**	***P*_trend_**	**HR (95% CI)**	***P*_trend_**	***P*_difference_**	**HR (95% CI)**	***P*_trend_**	**HR (95% CI)**	***P*_trend_**	***P*_difference_**
*Protein*	35	1.37 (0.95–1.99)	0.10	1.12 (0.64–1.94)	0.70	0.54	1.13 (0.76–1.70)	0.54	1.08 (0.65–1.79)	0.78	0.87
Animal protein^a^	35	1.23 (0.91–1.66)	0.17	0.95 (0.62–1.45)	0.82	0.33	0.92 (0.66–1.28)	0.63	1.03 (0.71–1.49)	0.90	0.67
Dairy protein^b^	35	1.22 (0.81–1.85)	0.34	1.28 (0.73–2.22)	0.39	0.90	1.10 (0.68–1.78)	0.69	1.76 (1.06–2.95)	0.03	0.19
Plant protein^a^	35	0.88 (0.42–1.84)	0.73	0.75 (0.27–2.10)	0.58	0.81	1.14 (0.53–2.43)	0.74	0.63 (0.24–1.67)	0.35	0.34
											
*Calcium* b	0.3	1.07 (0.96–1.19)	0.25	1.05 (0.91–1.22)	0.49	0.90	1.00 (0.88–1.14)	0.96	1.19 (1.04–1.37)	0.01	0.07
Dairy calcium^b^	0.3	1.06 (0.96–1.17)	0.28	1.04 (0.91–1.19)	0.57	0.86	1.01 (0.90–1.14)	0.82	1.16 (1.03–1.32)	0.02	0.12
Nondairy calcium^b^	0.3	1.14 (0.66–1.99)	0.63	1.04 (0.47–2.29)	0.93	0.85	0.83 (0.44–1.56)	0.57	1.21 (0.57–2.56)	0.62	0.46

All models are stratified by centre and adjusted for education, marital status, height, weight and energy intake.

a Greece and Umeå excluded due to missing data.

b Greece excluded due to missing data.
